# Anti-hepatitis, antioxidant activities and bioactive compounds of *Dracocephalum heterophyllum* extracts

**DOI:** 10.1186/s40529-016-0133-y

**Published:** 2016-08-06

**Authors:** Qiang-Qiang Shi, Jun Dang, Huai-Xiu Wen, Xiang Yuan, Yan-Duo Tao, Qi-Lan Wang

**Affiliations:** 1 Key Laboratory of Tibetan Medicine Research, Northwest Institute of Plateau Biology, CAS, Xining, China; 2 University of Chinese Academy of Science, Beijing, China

**Keywords:** Anti-hepatitis activity, Antioxidant activity, Chemical constituents, *Dracocephalum heterophyllum*

## Abstract

**Background:**

*Dracocephalum heterophyllum* was a traditional Tibetan medicine possesses various pharmacological effects involved in anti-inflammatory, antibacterial activities. However, its anti-hepatitis, antioxidant activity and bioactive compounds have not been reported, the objective of this research work was to investigate the pharmacological activity and bioactive compounds of *D. heterophyllum* extracts.

**Results:**

In the present study, the anti-hepatics and antioxidant activities of four *D. heterophyllum* extracts (i.e. petroleum ether extracts, ethyl acetate extracts, *n*-BuOH extracts, and water extracts) were conducted. The main chemical constituent of petroleum ether and ethyl acetate extracts were also isolated using chromatographic techniques and identified by NMR spectroscopic methods. The anti-hepatitis assay showed that the petroleum ether and ethyl acetate extracts of *D. heterophyllum* significantly prolonged the mean survival times and reduced the mortality of mouse hepatitis model induced by concanavalin A (ConA). The levels of alanine transaminase, aspartate transaminase in blood serum could be decreased obviously by ethyl acetate extracts compared with ConA group (P < 0.01). The histological analysis demonstrated that the ethyl acetate extracts could inhibit apoptosis and necrosis caused by ConA. In addition, the antioxidant activities of the four extracts of *D. heterophyllum* were measured by DPPH assay, ABTS assay, anti-lipidperoxidation assay, ferric reducing antioxidant power assay, ferrous metal ions chelating assay and determination of total phenolic contents. The results showed that the ethyl acetate extract had the highest antioxidant activities, followed by petroleum ether extract. Finally, nine mainly compounds were isolated from the Petroleum ether and ethyl acetate extracts, including four triterpenes: oleanolic acid (**1**), ursolic acid (**2**), pomolic acid (**3**), 2α- hydroxyl ursolic acid (**4**), three flavonoids: apigenin-7-*O*-rutinoside (**5**), luteolin (**8**), diosmetin (**9**) and two phenolic acids: rosmarinic acid (**6**), methyl rosmarinate (**7**).

**Conclusion:**

The Ethyl acetate extract of *D. heterophyllum* had the highest anti-hepatitis and antioxidants activities, followed by petroleum ether extract. The bioactive substances may be triterpenes, flavonoids and phenolic acids, the ethyl acetate extracts of *D. heterophyllum* may be possible candidates in developing anti-hepatitis medicine.

**Electronic supplementary material:**

The online version of this article (doi:10.1186/s40529-016-0133-y) contains supplementary material, which is available to authorized users.

## Background

Hepatitis is an inflammation of the liver. The condition can progress to fibrosis (scarring), cirrhosis or liver cancer. Hepatitis viruses are the most common cause of hepatitis in the world. There are five main hepatitis viruses, referred to as types A, B, C, D and E. In particular, types B and C lead to chronic disease in hundreds of millions of people and, together, are the most common cause of liver cirrhosis and cancer. More than 20 million people worldwide are infected with hepatitis virus. And infection from these viruses results in approximately 1.45 million deaths each year. Effective prophylactic vaccines have been available since the 1980s (Hollinger and Liang 2001). Nonetheless, for many developing countries, large-scale vaccination programs were hardly affordable, and an enormous number of chronic hepatitis virus carriers will be in need of better medication for decades to come. Current therapies are based on the systemic administration of high doses of interferon-α (IFN-α) or on nucleoside analogs. However, both therapies have a sustained response rate of only about 30 %, combinations exert no clear synergism, and lamivudine therapy leads to the rapid emergence of resistant virus variants (Pumpens et al. 2002; Zoulim 2001). Hepatitis B virus (HBV) is a hepadnavirus DNA virus with species specificity and tissue specificity, normally infect only humans and chimpanzees. There is no other feasible small experimental animal infection model. It is also very difficult to infect the cells cultured in vitro. For now, though there are many hepatitis animal model, such as duck hepatitis B model (Schultz et al. 2004), woodchuck model (Wang et al. 2004), chimpanzees model (Wieland et al. 2004), HBV transgenic mice model (Chisari 1995), there are still some different levels of flaws.

*Dracocephalum heterophyllum* is a traditional Tibetan medicine growing on Qinghai-Tibet Platean with special living environment of high elevation and strong sunlight irradiation. The plant is distributed widely in Sitsang, Qinghai, Sinkiang and Gansu province of China. In traditional Tibetan Medicine, *D. heterophyllum* is known as Ao-Ga or Ji-Mei-Qing-Bao, which has been used as an ethnomedicine to treat various ailments such as jaundice, hepatopathy, cough, lymphangitis, mouth ulcers and tooth diseases. There had been reports said that the herb had antiviral activity (Zhang et al. 2009), antianoxic effect (Peng 1984), antiasthmatic, anticoughing and disinfectant action (Mahmood et al. 2005), and the essential oil of it also had antimicrobial and antioxidant activities (Zhang et al. 2008).

The aim of this paper was to evaluate the anti-hepatitis activities of *D. heterophyllum* in the mouse fulminant hepatitis model induced by concanavalin A (ConA), and measured the antioxidant activities of this herbs in a series of in vitro assay such as free radical scavenging experiments (DPPH and ABTS assay), anti-lipidperoxidation experiments (FTC assay), ferric reducing antioxidant power assay (FRAP), metal chelating assay and determination of total phenolic contents (TPC). Finally, the bioactive substances were also separated and purified using chromatographic techniques.

## Methods

### Chemical reagent

Female Bal B/C mice were bought from Beijing Vitalriver Experimental Animals Ltd. (Beijing, China), The animals were performed according to guidelines laid down by the Animal Care and Use Committee of Shenzhen Institutes of Advanced Technology, Chinese Academy of Sciences (Approval IDs: SCXY2012-0119) that follows internationally acceptable standards on animal care and use in laboratory experimentation. Concanavalin A (ConA) was obtained from Sigma-Aldrich Co. (Shanghai, China), ALT kit, AST kit, TUNEL kit, DAPI kit were purchased from Nanjing Jiancheng Bioengineering Institute (Nanjing, China), 1,1-Diphenyl-2-picryl-hydrazyl (DPPH), 2-azino-bis(3-ethylbenzthiazoline-6-sulfonic acid) (ABTS), 3-(2-pyridyl)-5,6-bis (4-phenyl-sulfonic acid)-1,2,4-triazine (ferrozine), 2,4,6-Tris(2-pyridyl)-s-triazine (TPTZ), linoleic acid, α-Tocopherol, butylated hydroxytoluene (BHT), butylated hydroxyanisole (BHA), potassium persulfate (K_2_S_2_O_8_), ammonium thiocyanate were (NH_4_SCN), ferrous chloride tetrahydrate (FeCl_2_·4H_2_O), Ferric chloride tetrahydrate (FeCl_3_·6H_2_O), sodium tungstate dihydrate (Na_2_WO_4_·2H_2_O), sodium molybdate dehydrate (Na_2_MoO_4_·2H_2_O), sodium carbonate anhydrous (Na_2_CO_3_) were all purchased from Aladdin Industrial Corporation (Shanghai, China). Gallic acid was obtained from National Institute for Food and Drug Control (Beijing, China). All other chemical reagent and buffer used were analytical grade and obtained from Beijing Chemical Co. (Beijing, China).

### Sample preparation

The whole grass of *D. heterophyllum* was harvested in August 2014, from North Mountain in Huzhu, Qinghai province, China. The sample was identified by MEI Li-juan (Northwest Institute of Plateau Biology, Qinghai, China). The fresh samples were air-dried in hade, then ground into a homogeneous powder in a mill.

8.86 kg of air-dried and powered *D. heterophyllum* was extracted by 95 % ethanol at 70 °C through heating reflux. The samples were filtered with filter paper while the residue was further extracted under the same conditions 3 times. The filtrates were collected, and then ethanol was removed by a rotary evaporator (EYELA, Japan) at 50 °C to get the crude extract of *D. heterophyllum*.

The crude ethanol extract (1026 g) of *D. heterophyllum* was suspended into 500 mL water. The suspension was successively extracted 3 times by the same volume of petroleum ether, ethyl acetate and n-butanol at room temperature to get four fractions. Then the four fractions were dried by a rotary evaporator (EYELA, Japan), respectively. The four extracts were stored at 4 °C until used.

### Anti-hepatitis activity assay

#### The survival experiment of mice with lethal doses of ConA

Concanavalin A (ConA) is a plants lectin, and is known for its ability to stimulate mouse T cell subsets giving rise to four functionally distinct T-cell populations, including precursors to suppressor T-cell; one subset of human suppressor T-cells as well is sensitive to ConA (Dwyer and Johnson 1981). Female BALB/c mice would develop severe liver injury as assessed by transaminase release within 8 h when an intravenous dose concanavalin A (Con A) was given. Histopathologically, only the liver was affected. Con A-induced liver injury depends on the activation of T lymphocytes by macrophages in the presence of ConA (Tiegs et al. 1992). The model might allow the study of the pathophysiology of autoimmune hepatitis and viral hepatitis.

Female Balb/C mice 9–10 weeks old weighing about 25–29 g were housed for 1 day to acclimatize. The mice were randomly divided into nine groups each comprised of eight mice. The first group, ConA group, was only injected with 1 mg/mL ConA in caudal vein at the lethal dose of 20 mg/Kg. The next four groups, drug group, were only injected with 10 mg/mL four extracts of *D. heterophyllum* in abdominal cavity at the dose of 50 mg/Kg, respectively. The last four groups, ConA+ drug group, were injected with 10 mg/mL four extracts of *D. heterophyllum* in abdominal cavity at the dose of 50 mg/Kg, respectively, and 2 h later, were injected with 1 mg/mL ConA in caudal vein at the dose of 20 mg/Kg to induced hepatitis. The mice were fed and observed for 24 h to determine their mortality.

#### The level of transaminase in mice serum

According to the (Table 1), 32 female Balb/C mice were randomly divided into four groups each comprised of eight mice. The first group, control group, was only injected with DMSO and PBS in abdominal cavity. The second group, ConA group, was only injected with 1 mg/mL ConA in caudal vein at the dose of 12.5 mg/Kg. The third group, drug group, was only injected with 10 mg/mL Ethyl acetate extracts of *D. heterophyllum* in abdominal cavity at the dose of 50 mg/Kg. The last group, ConA+ drug group, were injected with 10 mg/mL ethyl acetate extracts of *D. heterophyllum* in abdominal cavity at the dose of 50 mg/Kg, and 2 h later, was injected with 1 mg/mL ConA in caudal vein at the dose of 12.5 mg/Kg. The blood was taken using orbital venous plexus blood collection in the time of 8, 16, 24 h after injected ConA. The blood volume was collected 200–300 μL, and centrifuged 5 min at 3000 rpm. The upper serum was moved to the new Eppendorf tube, and saved at −20 °C, temporarily. The ALT and AST kit were used for quantitation of transaminase in mice serum of different groups.

**Table 1:** The treatment on the hepatitis model induced by conA

Groups	The number of mice	Method of administration	Testing index
Control group	8	Abdominal cavity	ALT/AST
ConA group	8	Caudal vein	ALT/AST
Drug group	8	Abdominal cavity	ALT/AST
ConA+ drug group	8	Caudal vein + abdominal cavity	ALT/AST

*ConA* concanavalin A; *ALT* alanine transferase; *AST* aspartate transaminase; *Drug* ethyl acetate extracts of *D. heterophyllum*

### Morphologic, histological and hepatocytes apoptosis analysis

Pretreating the mice base on the (Table 1). Test mice breaking the neck to death after induced by ConA 24 h later, removing the liver, observing the hepatic pathological changes of liver using morphologic method. Then the livers were fixed in 10 % formalin, embedded in paraffin and cut into slices. HE staining were performed and the result of the staining was analyzed with microscopic examination. The method of TdT mediated dUTP nick end labeling (TUNEL) and 4′,6-diamidino-2-phenylindole (DAPI) were used to detect apoptosis of mouse liver.

### Antioxidant activity

#### DPPH free radical scavenging activity

The free radical scavenging activity of four *D. heterophyllum* extracts were estimated using in vitro 1,1-diphenyl-2-picryl-hydrazil (DPPH) free radical assay following the methodology described by Blois (1958). DPPH free radical reagent solution was prepared by dissolving DPPH in ethanol to obtain 0.1 mM concentration. Four extracts test solution were prepared by dissolving different concentrations of four extracts (10, 50, 100, 150 200, 300, 500 μg/mL) in ethanol. To 2 mL of test solution 2 mL of freshly prepared DPPH reagent was added. The reaction mixture was incubated in dark at room temperature. Thirty minutes later, the absorbance was measured at 517 nm using 752 N UV–Vis spectrophotometer (Shanghai, China). Aliquots of α-tocopherol, BHT and BHA was served as standards for the assay. A mixture of DPPH and ethanol was served as control and a mixture of *D. heterophyllum* extracts solution and ethanol was served as a blank for the sample. All experiments were repeated three times and take the mean to eradicate any discrepancies. % inhibition of DPPH was calculated by the following formula:

$${\text{Inhibition of DPPH }}\left( \% \right) \, = \, \left[ {{{\left( {{\text{A}}_{\text{Control}} - {\text{A}}_{\text{Sample}} } \right)} / {{\text{A}}_{\text{Control}} }}} \right] \, \times { 1}00$$

where A_Control_ is the absorbance of the control reaction, while A_Sample_ is the absorbance at 517 nm with *D. heterophyllum* extracts.

#### ABTS free radical scavenging activity

The ABTS·^+^ free radical scavenging activity was measured as the methodology described by Li et al. (2012) with some modifications. The ABTS·^+^ was produced by mixing 0.2 mL ABTS diammonium salt (7.4 mM) with 0.2 mL potassium persulfate (2.6 mM). The mixture was kept in the dark at room temperature for 12 h to allow completion of radical generation, then diluted with 95 % ethanol (about 40–50 times) so that its absorbance at 734 nm was 0.70 ± 0.02. To determine the scavenging activity, 4 mL aliquot of ABTS·^+^ reagent was mixed with 1 mL of sample alcoholic solutions (10–500 μg/mL). After incubation for 6 min, the absorbance at 734 nm was read on 752 N UV–Vis spectrophotometer (Shanghai, China). Aliquots of α-tocopherol, BHT and BHA was served as standards for the assay. A mixture of ABTS·^+^ reagent and ethanol was served as control and a mixture of *D. heterophyllum* extracts solution and ethanol was served as a blank for the sample. All experiments were repeated three times and take the mean to eradicate any discrepancies. The percentage inhibition of the samples was calculated as:

$${\text{Inhibition of ABTS}} \cdot^{ + } \left( \% \right) \, = \, \left[ {{{\left( {{\text{A}}_{\text{Control}} - {\text{ A}}_{\text{Sample}} } \right)} / {{\text{A}}_{\text{Control}} }}} \right] \, \times { 1}00$$

where A_Control_ is the absorbance of the control reaction, while A_Sample_ is the absorbance at 734 nm with *D. heterophyllum* extracts.

### Anti-lipidperoxidation activity by FTC

The anti-lipidperoxidation activity of four *D. heterophyllum* extracts and standards was measured by ferric thiocyanate method (Kikuzaki and Nakatani 1993), which was slightly modified. A mixture of 1 mL of sample alcoholic solutions (25 and 50 μg/mL), 1 mL of 2.5 % linoleic acid solution in ethanol, 2 mL of a 0.05 M phosphate buffer (pH 7.0) and 1 mL of distilled water was placed in a centrifuge tube (15 mL) with a screw cap. The mixed solution (5 mL) was incubated in an oven at 40 °C in the dark. On the other hand, the 5 mL control was composed of 1 mL of ethanol, 1 mL of 2.5 % linoleic acid solution in ethanol, 2 mL of a 0.05 M phosphate buffer (pH 7.0) and 1 mL of distilled water. To 0.1 mL of this solution was added 9.5 mL of 75 % ethanol and 0.1 mL of 30 % ammonium thiocyanate. Exactly 3 min after adding 0.1 mL of 0.02 M FeCl_2_ in 3.5 % HCl, the absorbance of red color was measured at 500 nm. This step was repeated every 24 h until 1 day after absorbance of the control reached maximum. The percentage inhibition of lipid peroxidation in linoleic acid emulsion was calculated by following equation.

$${\text{Inhibition of lipid peroxidation }}\left( \% \right) \, = \, \left[ {{{\left( {{\text{A}}_{\text{Control}} - {\text{ A}}_{\text{Sample}} } \right)} / {{\text{A}}_{\text{Control}} }}} \right] \, \times { 1}00$$

where A_Control_ is the absorbance of the control reaction and A_Sample_ is the absorbance at 500 nm with *D. heterophyllum* extracts or standards compounds.

### Ferric reducing antioxidant power assay (FRAP)

The ability to reduce ferric ions was measured according to a modified method developed by Benzie and Strain (1996). To prepare the FRAP reagent, 0.1 mM acetate buffer (pH 3.6), 10 mM 2,4,6-tripyridyl-s-triazine (TPTZ) in 40 mM HCl, and 20 mM FeCl_3_·6H_2_O were mixed together in a ratio of 10:1:1 (v/v/v). A 100 μL aliquot of sample solutions (100–500 μg/mL) were added to 3 mL freshly prepared FRAP reagent. The absorbance of the reaction mixture was measured at 593 nm after 10 min incubation at 37 °C. Experiments were performed in triplicate. Aliquots of α-tocopherol, BHT and BHA was served as standards for the assay. The FeSO_4_·7H_2_O solutions (0.2–2 mM/L) was used to performed the calibration curves. The ferric reducing antioxidants power were calculated from the linear calibration curve and expressed by FRAP value: 1FRAP value = 1 mmol/L FeSO_4_.

#### Ferrous metal ions chelating activity

The ferrous metal chelating activity of four *D. heterophyllum* extracts and standards was estimated by the method of Dinis et al. (1994). Where in the ferrous ion chelating activity was measured by the absorbance of the ferrous iron-ferrozine complex at 562 nm. Briefly, four *D. heterophyllum* extracts (100–500 μg/mL) in 0.4 mL were added to 2 mM FeCl_2_ solution (0.05 mL), then 5 mM ferrozine (0.2 mL) was added in. The mixed solution was adjusted to 4 mL with ethanol. The reaction mixture was incubated in dark at room temperature for 10 min. Absorbance of the mixture was then measured at 562 nm. All tests and analyses were run in triplicate and averaged. The percentage of inhibition of the ferrozine–Fe^2+^ complex formation was calculated by the following formula (Gulcin 2006):

$${\text{Ferrous ions chelating effect }}\left( \% \right) \, = \, \left[ {{{\left( {{\text{A}}_{\text{Control}} - {\text{ A}}_{\text{Sample}} } \right)} \mathord{\left/ {\vphantom {{\left( {{\text{A}}_{\text{control}} - {\text{ A}}_{\text{sample}} } \right)} {{\text{A}}_{\text{control}} }}} \right. \kern-0pt} {{\text{A}}_{\text{Control}} }}} \right] \, \times { 1}00$$

where A_Control_ is the absorbance of the control reaction, while A_Sample_ is the absorbance at 562 nm with *D. heterophyllum* extracts. The control contains FeCl_2_ and ferrozine, complex formation molecules.

#### Determination of total phenolic contents (TPC)

The total phenolic contents of four *D. heterophyllum* extracts were determined using the Folin-Ciocalteu method (Ragazzi and Veronese 1973) with a little modification. To prepare the Folin-Ciocalteu reagent, 25 g sodium tungstate, 6.25 g sodium molybdate, 175 mL distilled water, 8.5 % phosphoric acid solution, and 25 mL concentrated hydrochloric acid were mixed together. The above mixture was refluxed slowly in a water bath for 10 h. After cooling, 37.5 g lithium sulfate, 12.5 mL distilled water, and 50 mL hydrogen peroxide were added in, then continued to heat in boiling water for 15 min without cap. Finally dilute with water to 250 mL, and stored at 4 °C until used. 100 μL sample solution (1 mg/mL) was mixed with 500 μL Folin-Ciocalteu reagent and diluted with 1000 μL distilled water, then 1.5 mL of Na_2_CO_3_ solution (20 %, w/v) was added. Absorbance of the mixture was measured at 765 nm after 2 h incubation in dark at home temperature. The determinations were performed in triplicate, and the calculations were based on a calibration curve obtained with gallic acid (100–800 μg/mL).

#### Separation and purification of the bioactive compounds

The petroleum ether extract (500 g) was subjected to a silica gel column (petroleum ether-acetone, 95:5–5:95) to afford fractions (I–VI). Fraction III (150 g), the mixture of two compounds, was purified on a preparative C_18_ HPLC column with a isocratic of MeOH–H_2_O (87:13) to yield compound **1** (3 mg) and compound **2** (33 mg). The two compounds were difficult to separate. Fraction IV was purified on a preparative C_18_ HPLC column with a gradient of C_2_H_3_N–H_2_O (65:35–95:5) to yield compound **3** (130 mg) and compound **4** (75 mg). Fraction VI was purified on a preparative C_18_ HPLC column with a gradient of C_2_H_3_N–H_2_O (20:80–30:70) to yield compound **5** (200 mg).

The ethyl acetate extract (19 g) was subjected to medium pressure liquid chromatography with a gradient of MeOH–H_2_O (3:7–8:2) to afford fractions (I–VII). Fraction III (3 g) was purified on a preparative C_18_ HPLC column with a gradient of C_2_H_3_N−H_2_O (10:90–30:70) to yield compound **6** (1.5 g). Fraction IV (2.3 g) was purified on a preparative C_18_ HPLC column with a isocratic of C_2_H_3_N−H_2_O (25:75) to yield compound **7** (853 mg). Fraction V (1.1 g) was purified on a preparative C_18_ HPLC column with a isocratic of C_2_H_3_N−H_2_O (28:72) to yield compound **8** (58 mg) Fraction IV (1.1 g) was purified on a preparative C_18_ HPLC column with a isocratic of C_2_H_3_N–H_2_O (27:73) to yield compound **9** (87.9 mg).

## Results and discussion

### Extraction and fractionation of *Dracocephalum heterophyllum*

From 8.86 kg of *D. heterophyllum*, 1026 g of 95 % ethanol extract was obtained. The yield was 11.6 %. The crude extract (1026 g) was then suspended in water and extracted with petroleum ether, ethyl acetate and *n*-butanol sequentially to get 500 g petroleum ether fraction (48.7.6 %), 19 g ethyl acetate fraction (2 %), 41.8 g *n*-butanol fraction (4.1 %) and 115.3 g water fraction (11.2 %).

### Anti-hepatitis activity

#### The survival experiment of mice with lethal doses of ConA

**Fig. 1:**
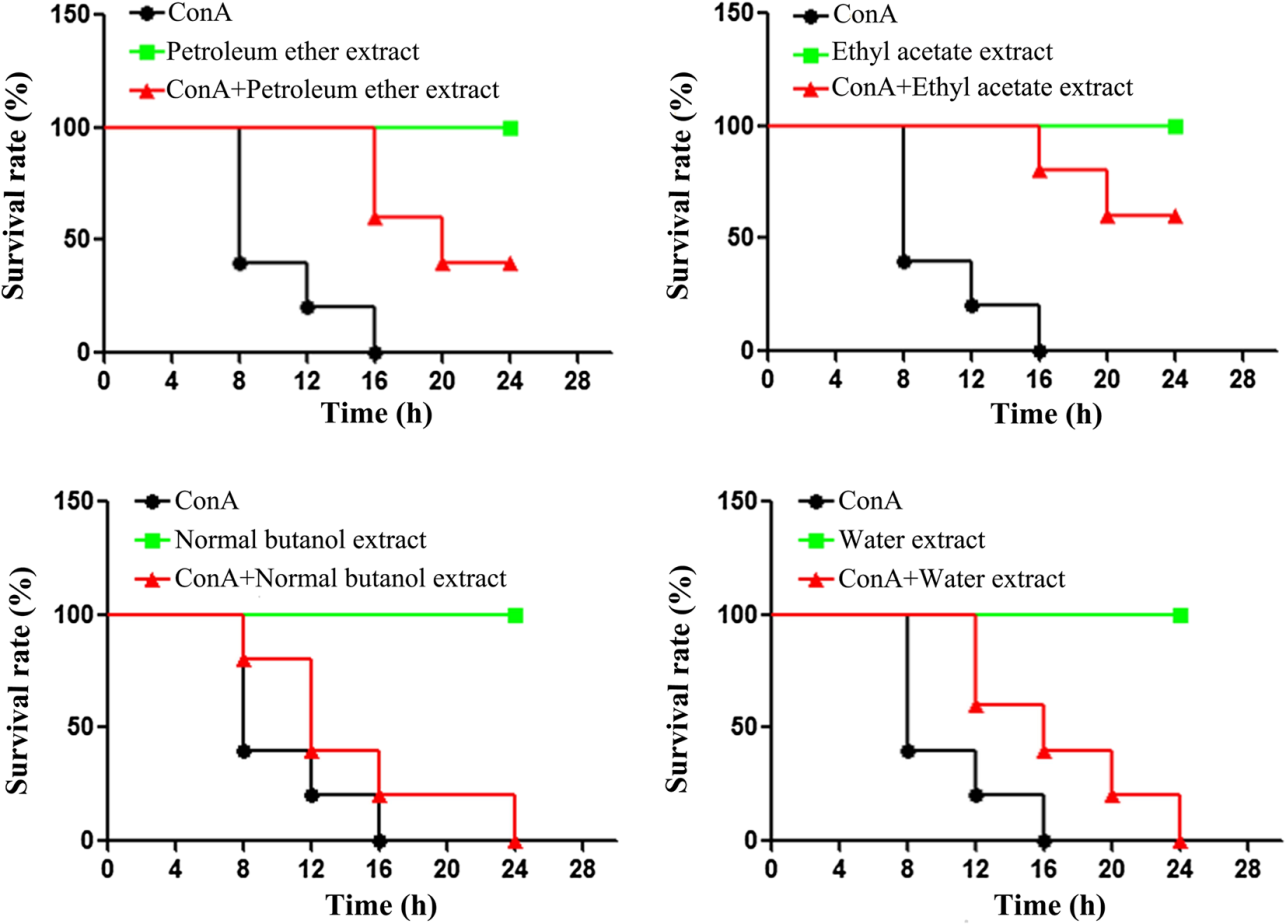
The experiments of survival rate. Female Balb/C mice were injected with four extracts of *D. heterophyllum* (50 mg/kg) at 2 h before the injection of a lethal dose of Con A (20 mg/kg). The survival rate was monitored at different times after Con A administration

As show in Fig. 1, the survival rate of mice in the first group, ConA group, is lower than 50 % in 8 h, and the mice were all die in 16 h. The mice in the second group, drug group, were all still living in 24 h. Its means that the four extracts of *D. heterophyllum* have no toxic effect on mice. The survival time of mice in the third group, ConA+ drug group, is all longer than ConA group on different levels, respectively. In particular, the survival rate of mice which were injected with petroleum ether extract is nearer to 50 % in 24 h. And the survival rate of mice which were injected with ethyl acetate extract has beyond 65 % in 24 h. The experimental results suggest, the petroleum ether and ethyl acetate extracts of *D. heterophyllum* significantly prolonged the mean survival times and reduced the mortality of ConA induced mice.

#### The level of transaminase in mice serum

**Fig. 2:**
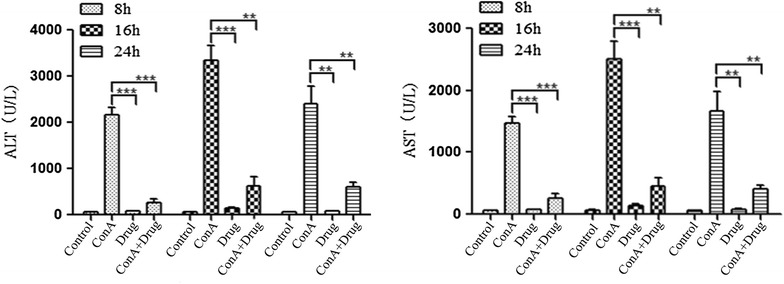
The level of ALT and AST in mice serum. Female Balb/C mice were injected with ethyl acetate extract of *D. heterophyllum* (50 mg/kg) at 2 h before the injection of a dose of Con A (12.5 mg/kg). Serum transaminase ALT and AST levels were determined 8, 16, 14 h after the Con A injected. Data expressed as mean ± SD (n = 8; **P < 0.01 and ***P < 0.001)

In biochemistry, a transaminase or an aminotransferase is an essential enzyme in the process of normal cellular metabolism, and mainly remains in hepatocytes. In medicine, the presence of elevated transaminases in serum can be an indicator of liver damage. Two important transaminase enzymes are alanine transaminase (ALT), also called alanine aminotransferase (ALAT) or serum glutamate-pyruvate transaminase (SGPT); and aspartate transaminase (AST), also known as serum glutamic oxaloacetic transaminase (SGOT). As show in Fig. 2, the level of ALT and AST in ConA group was significantly higher than control group (P < 0.01). Its means that ConA can induce live damage, increase the contents of ALT and AST in serum. But, the level of ALT and AST in ConA+ drug group was obviously lower than ConA group (*P* < 0.01). Its suggest that ethyl acetate extract of *D. heterophyllum* can improve live injury induced by ConA.

### Morphologic, histological and hepatocytes apoptosis analysis

**Fig. 3:**
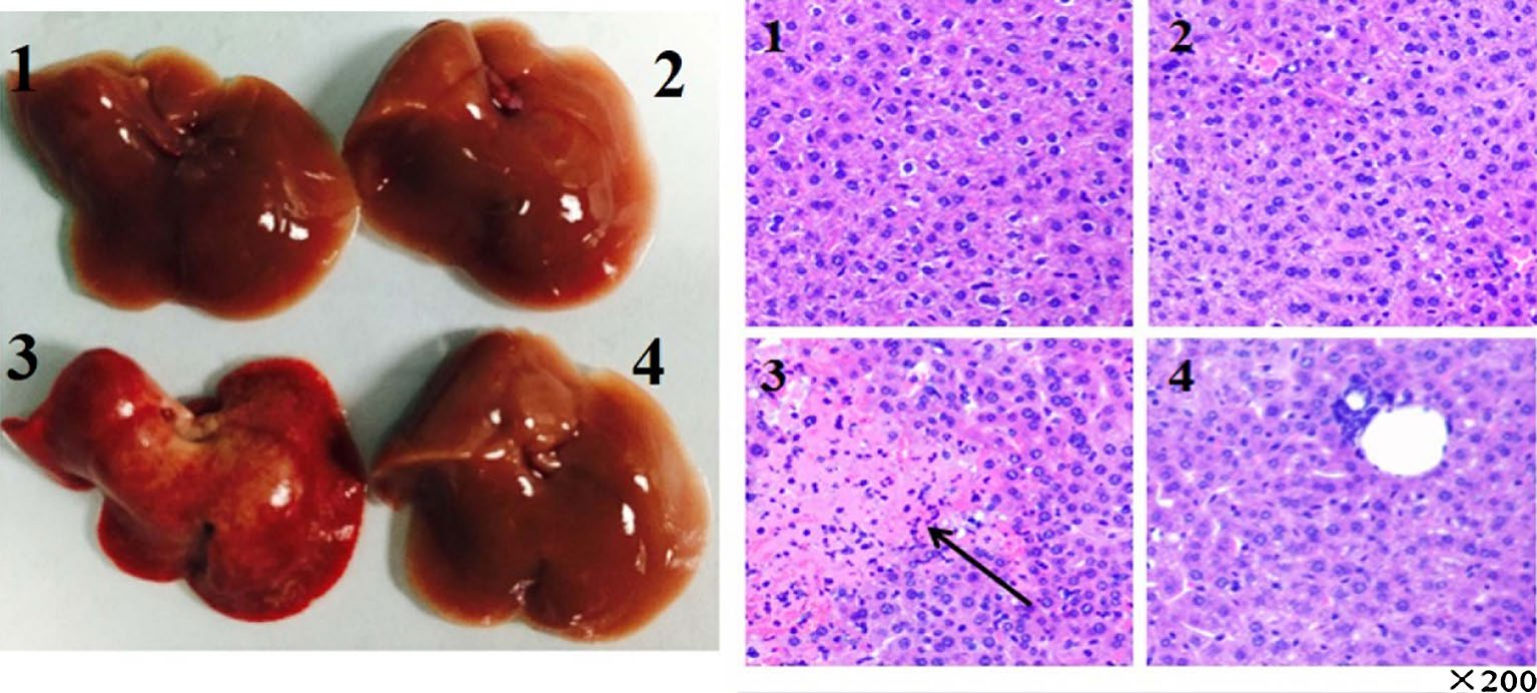
Morphological change and histological pathologic qualitative evaluation for the livers of experimental group and controlled group. Mice were sacrificed at 24 h after the Con A injection. The liver were harvested from control (*1*), drug (*2*), Con A (*3*) and Con A + drug (*4*) mice respectively. And the liver tissues of the four groups were fixed and strain with hematoxylin and eosin (H and E). The arrow indicates massive cell death in the liver section. Original magnification ×200

As show in Fig. 3, in contrast with control group, the liver of ConA group had serious pathological changes, the liver issue become harden, the color become deepen, external surface of liver studded with a lot of regenerated nodules. Meanwhile the pathological histology analysis results showed that inflammatory cellular infiltration and necrosis were observed in ConA group. However, compared with ConA group the liver lesions of the ConA+ drug group were improved dramatically, and the inflammatory cells and necrosis of hepatic cells were significantly reduced.

**Fig. 4:**
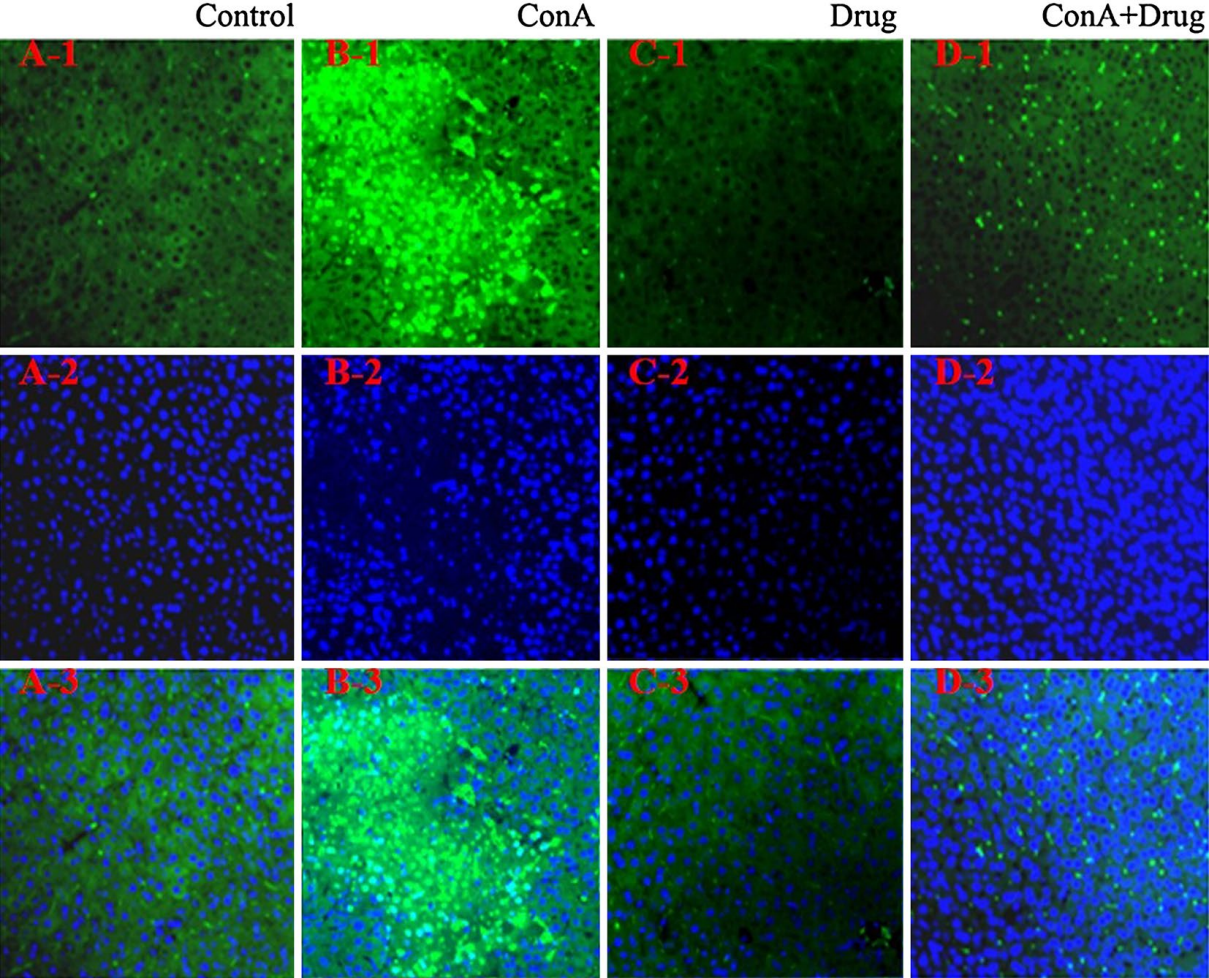
Analysis of liver cell apoptosis. Mice were sacrificed at 24 h after Con A injection. The livers were harvested from control (**A**), Con A (**B**), drug (**C**) and ConA+ drug (**D**) groups respectively. Liver tissues from the four groups were fixed and stained with TUNEL (1), DAPI (2) and overlap (3). Original magnification ×200

The result of TUNEL assay indicated that there were many apoptotic cells in the ConA group compared with control group. And the DAPI staining showed nuclear condensation and fragmentation in ConA group. But, in ConA+ drug group, there were only A few apoptotic cells were detected. The results suggested that ConA can induce a large amount of apoptotic cells, and the ethyl acetate extracts of *D. heterophyllum* can improve apoptotic (Fig. 4).

Liver damage caused by hepatitis is associated with excessive activation of the immune responses. It has been reported that TNF-α participate in various forms of liver damage, such as viral, toxic and autoimmune hepatitis, and play an important role in ConA-induced hepatitis. It has also been reported that Kupffer cells secrete a large amount of TNF-α to aggravate the liver damage. Kupffer cells play an important role in T cell activation-induced liver injury. However, the mechanism may not be that simple, and more research is needed.

### Antioxidant activity

#### DPPH free radical scavenging activity

**Fig. 5:**
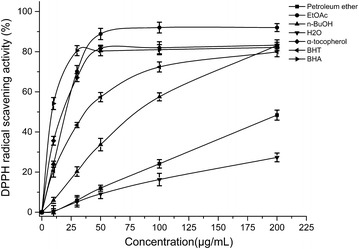
Free radical scavenging activity of various fractions of *D. heterophyllum*. 0, 10, 30, 50, 100, 200 μg/mL of four *D. heterophyllum* extracts were measured by the DPPH method at 517 nm. BHT, BHA and α-tocopherol were used as standard antioxidants

In this study, the antioxidant activities of four *D. heterophyllum* extracts and standards such as α-tocopherol, BHT and BHA were determined using the DPPH coloring method in different concentrations (10–500 μg/mL). Figure 5 illustrates the radical-scavenging activity of the different fractions of *D. heterophyllum* and three standards antioxidants. The scavenging effect of four *D. heterophyllum* extracts and standards on the DPPH radical decreased in the order: EtOAc > α-tocopherol > BHT > BHA > *n*-BuOH > petroleum ether > water, which was 88.8, 81.6, 80.4, 57.3, 33.7, 12, 9.1 % at a concentration of 50 µg/mL, respectively. Figure 5 also illustrated that the free radical scavenging activity of these samples increased with increasing concentration obviously.

#### ABTS·^+^ free radical scavenging activity

**Fig. 6:**
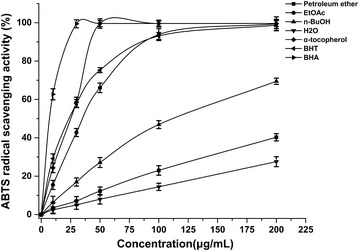
Free radical scavenging activity of various fractions of *D. heterophyllum*. 0, 10, 30, 50, 100, 200 μg/mL of four *D. heterophyllum* extracts were measured by the ABTS method at 734 nm. BHT, BHA and α-tocopherol were used as standard antioxidants

In this experiment, BHT, BHA and α-tocopherol were served as standard antioxidants. As seen in (Fig. 6), the extracts of *D. heterophyllum* had effective ABTS·^+^ free radical scavenging activity, and the scavenging activity of four fractions of *D. heterophyllum* and standards antioxidants on the ABTS·^+^ decreased in that order: α-tocopherol = BHA > BHT > EtOAc > n-BuOH > petroleum ether > water, which were 99.56, 99.56, 75.2, 66.2, 27.1, 12.2 and 8.0 %, at the concentration of 50 μg/mL, respectively.

### Anti-lipidperoxidation activity

**Fig. 7:**
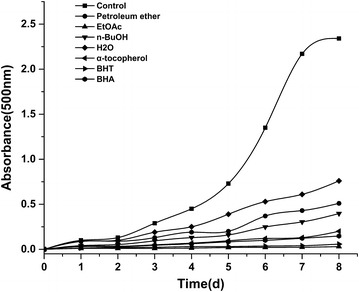
Anti-lipidperoxidation activity of various fractions of *D. heterophyllum* (50 μg/mL) was measured by the FTC method at 500 nm. BHT, BHA and α-tocopherol were used as standard antioxidants

The petroleum ether fraction, EtOAc fraction, *n*-BuOH fraction, water fraction of *D. heterophyllum* and standards compounds exhibited effective Anti-lipidperoxidation activity. At the concentration of 50 μg/mL, the effects of four fractions of *D. heterophyllum* and α-tocopherol, BHT and BHA on lipid peroxidation of the linoleic acid emulsion are shown in (Fig. 7). The percentage inhibition of peroxidation in the linoleic acid system by 50 µg/mL concentrations of petroleum ether fraction, EtOAc fraction, *n*-BuOH fraction and water fraction of *D. heterophyllum* was found to be 78.2, 98.7, 83.0 and 67.5 %, respectively. On the other hand, the percentage inhibition of 50 µg/mL concentrations of standards α-tocopherol, BHT and BHA was found to be 91.5, 97.5 and 93.75 %, respectively. The result showed that the EtOAc fraction of *D. heterophyllum* had the highest anti-lipidperoxidation activity.

### Ferric reducing ability of plasma (FRAP)

The FeSO_4_·7H_2_O solutions (0.2–2 mM/L) was used to performed the calibration curves, regression equations of it was Y = 0.68006X + 0.00916, R^2^ = 0.999. The ferric reducing antioxidant power of four *D. heterophyllum* extracts and standards antioxidants were expressed as FRAP value: 1 FRAP value = 1 mmol/L FeSO_4_.

**Fig. 8:**
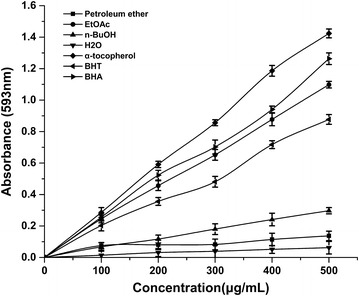
Linearity of FRAP: dose–response lines for solutions of four *D. heterophyllum* extracts. BHT, BHA and α-tocopherol were used as standard antioxidants

α-tocopherol, BHA and BHT had the reducing antioxidant power of 2.1 ± 0.01, 1.8 ± 0.01 and 1.3 ± 0.02 FRAP value. The EtOAc fraction had highest reducing antioxidant power with 1.6 ± 0.01 FRAP value, and followed by petroleum ether, *n*-BuOH and water fraction. The (Fig. 8) illustrates the dose response of each individual antioxidant tested was linear, showing that reducing antioxidant activity is not concentration-dependent, at least over the concentration ranges tested in this study.

#### Ferrous metal ions chelating activity

**Fig. 9:**
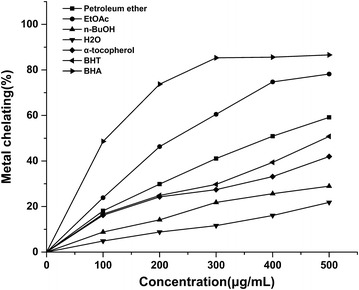
Ferrous ions chelating effect of different concentrations of Petroleum ether fraction, EtOAc fraction, *n*-BuOH fraction, water fraction α-tocopherol, BHT and BHA

As shown in (Fig. 9), the metal chelating activities of petroleum ether fraction, EtOAc fraction, *n*-BuOH fraction, water fraction and standards were concentration-dependent. The difference between the four *D. heterophyllum* factions and the control was statistically significant (P < 0.01). In addition, the percentages of metal scavenging capacity of 0.5 mg/mL concentration of Petroleum ether fraction, EtOAc fraction, *n*-BuOH fraction, water fraction, α-tocopherol, BHT and BHA were found as 59.2, 78.2, 29.0, 21.8, 42.0, 58.8, 86.6 %, respectively. These results show that the ferrous ion chelating effect of EtOAc fraction was statistically similar to BHA (P > 0.05) but higher than BHT (P < 0.05) and α-tocopherol (P < 0.05), the petroleum ether fraction was similar to BHT (P > 0.05), but higher than α-tocopherol (p < 0.05), the *n*-BuOH and water fraction had the lowest Ferrous metal ions chelating activity.

#### Total phenolic contents of fractions of *Dracocephalum heterophyllum*

By way of the literature review (Li et al. 2012), total phenolic was of significant positive correlations with antioxidant levels. In this paper, the total phenolic contents of petroleum ether, EtOAc, *n*-BuOH and water fractions were measured by the Folin-Ciocalteu reagent. The gallic acid (100–800 μg/mL) was used to performed the calibration curves, the regression equations of it was Y = 0.001X + 0.1693, R^2^ = 0.9957, and the total phenolic contents was expressed as Gallic acid mg/g of dry material. The results showed that EtOAc fraction had the highest phenolic contents of 433.7 mg gallic acid/g, *n*-BuOH and petroleum ether fraction had the phenolic contents of 110.7 and 70.7 mg/g. The water fraction had the lowest phenolic contents of 31.7 mg Gallic acid/g of dry material. This results indirectly reflect the antioxidant activities of *Dracocephalum heterophyllum* extracts.

The correlation of total phenolic contents (TPC) with DPPH assay (R^2^ = 0.9637, P < 0.05), ABTS assay (R^2^ = 0.9638, P < 0.05), FTC assay (R^2^ = 0.8203, P < 0.05) and FRAP assay (R^2^ = 0.9991, P < 0.05), respectively. The results demonstrated that the antioxidant activities of *D. heterophyllum* extracts had high correlation with total phenolic contents (TPC).

#### The chemical constituent of *Dracocephalum heterophyllum*

**Fig. 10:**
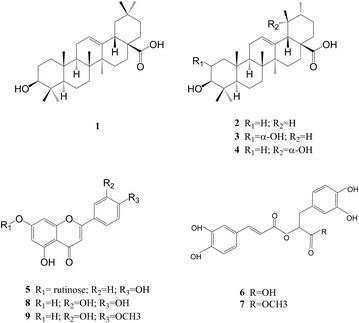
The compounds of *D. heterophyllum* extracts

The main chemical constituent of petroleum ether and ethyl acetate extracts were also isolated using chromatographic techniques and identified by NMR spectroscopic methods. 5 compounds were obtained from petroleum ether extract and 4 compounds were obtained from Ethyl acetate extract. These compounds were identified as oleanolic acid (**1**) (Seebacher et al. 2003), ursolic acid (**2**) (Seebacher et al. 2003), pomolic acid (**3**) (Cheng and Cao 1992), 2α- hydroxyl ursolic acid (**4**) (Kuang et al. 1989) and Apigenin-7-O-rutinoside (**5**) (Baris et al. 2011; Wang et al. 2003) from petroleum ether extracts, rosmarinic acid (**6**) (Kuhnt et al. 1994), methyl rosmarinate (**7**) (Kohda et al. 1989), luteolin (**8**) (Geiger et al. 1995; Xu et al. 2014) and diosmetin (**9**) (Sahu et al. 1998) from ethyl acetate extracts. The compound **1**–**4** were triterpenes, the compound **5**, **8** and **9** were flavonoids, the compound **6** and **7** were phenolic acids (Fig. 10). The ^1^H and ^13^C NMR data are as in Additional file 1. The main chemical constituents were isolated and identified, but the anti-hepatitis, antioxidants activities of these compounds require further study.

## Conclusions

In this paper, the mouse fulminant hepatitis model induced by ConA was first used to research the anti-hepatitis activity of petroleum ether, ethyl acetate, n-butyl alcohol and water extracts of *D. heterophyllum*. The antioxidant activity was also studied by some experiment in vitro, and the bioactive substances were isolated using chromatographic techniques and identified by NMR spectroscopic methods. Our results indicated that the ethyl acetate extract of *D. heterophyllum* had the highest anti-hepatitis and antioxidants activities, followed by the petroleum ether extract. The bioactive substances may be triterpenes, flavonoids and phenolic acids, the ethyl acetate extracts of *D. heterophyllum* may be possible candidates in developing anti-hepatitis medicine.

## Additional file

**Additional file 1.** NMR data.
