# Supplementary material for: Anti-hepatitis, antioxidant activities and bioactive compounds of Dracocephalum heterophyllum extracts
Source: Bot Stud. 2016 Aug 6;57:16. doi: 10.1186/s40529-016-0133-y (PMC5432916; doi:10.1186/s40529-016-0133-y)
Supplement: Supplementary file 1 — Additional file 1. NMR data. [file 40529_2016_133_MOESM1_ESM.doc]

Anti-hepatitis, antioxidant activities and bioactive compounds of *Dracocephalum heterophyllum* extracts

Qiang-Qiang SHI^a,b^, Jun DANG^a^, Huai-Xiu WEN^a^, Xiang YUAN^a,b^, Yan-Duo TAO^a^, and Qi-Lan WANG^a*^

**Oleanolic acid (1)**, white powder. ^1^H NMR(600 MHz, CD_3_OD) *δ*: 3.15(1H, dd, *J*=11.5, 4.6 Hz, H-3), 5.24(1H, t, *J*=3.3 Hz, H-12), 2.85(1H, dd, *J*=14.1, 4.6 Hz, H-18), 1.16(3H, s, H-23), 0.82(3H, s, H-24), 0.78(3H, s, H-25), 0.91(3H, s, H-26), 0.94(3H, s ,H-27), 0.97(3H, s, H-29), 0.95(3H, s, H-30); ^13^C NMR(150 MHz,CD3OD) *δ*: 39.8(C-1), 27.9(C-2), 79.7(C-3), 39.8(C-4), 56.8(C-5), 19.5(C-6), 34.0(C-7), 40.6(C-8), 49.6(C-9), 38.2(C-10), 24.0(C-11), 123.7(C-12), 145.2(C-13), 42.8(C-14), 28.7(C-15), 24.5(C-16), 47.6(C-17), 42.7(C-18), 47.2(C-19), 31.6(C-20), 34.9(C-21), 33.6(C-22), 28.9(C-23), 16.3(C-24), 15.9(C-25), 17.7(C-26), 26.4(C-27), 181.8(C-28), 33.8(C-29), 24.0(C-30).

**Ursolic acid (2)**, white powder. ^1^H NMR(600 MHz, CD_3_OD) *δ*: 3.15(1H, dd, *J*=11.6, 4.6 Hz, H-3), 5.23(1H, t, *J*=3.7 Hz, H-12), 2.20(1H, dd, *J*=11.4, 1.8 Hz, H-18), 1.12(3H, s, H-23), 0.85(3H, s, H-24), 0.78(3H, s, H-25), 0.97(3H, s, H-26), 0.96(3H, s, H-27), 0.89(3H, d, *J*=6.5 Hz, H-29), 0.98(3H, s, H-30); ^13^C NMR(150 MHz, CD_3_OD) *δ*: 39.8(C-1), 27.9(C-2), 79.7(C-3), 40.4(C-4), 56.7(C-5), 19.5(C-6), 34.3(C-7), 40.8(C-8), 49.6(C-9), 38.1(C-10), 24.1(C-11), 126.9(C-12), 139.6(C-13), 43.2(C-14), 28.7(C-15), 25.3(C-16), 48.1(C-17), 54.4(C-18), 40.4(C-19), 40.0(C-20), 31.8(C-21), 38.1(C-22), 29.2(C-23), 16.4(C-24), 16.0(C-25), 17.7(C-26), 24.4(C-27), 181.7(C-28), 17.8(C-29), 21.6(C-30).

**Pomolicacid (3)**, white powder.^1^H NMR(600 MHz, CD_3_OD) *δ*: 3.15(1H, dd, *J*=11.5, 4.7 Hz, H-3), 5.28(1H, t, *J*=3.8 Hz, H-12), 2.58(1H, td, *J*=13.3, 4.6 Hz, H-15), 2.50(1H, s, H-18), 0.98(3H, s, H-23), 0.8(3H, s, H-24), 0.78(3H, s, H-25), 0.95(3H, s, H-26), 1.33(3H, s, H-27), 0.93(3H, d, *J*=6.7 Hz, H-29), 1.19(3H, s, H-30); ^13^C NMR(150 MHz, CD_3_OD) *δ*: 39.8(C-1), 27.9(C-2), 79.8(C-3), 39.9(C-4), 56.8(C-5), 19.6(C-6), 34.2(C-7), 41.0(C-8), 48.6(C-9), 38.1(C-10), 24.8(C-11), 129.5(C-12), 139.9(C-13), 43.1(C-14), 29.6(C-15), 26.6(C-16), 49.6(C-17), 55.1(C-18), 73.6(C-19), 42.6(C-20), 27.1(C-21), 39.0(C-22), 28.8(C-23), 15.9(C-24), 16.3(C-25), 17.5(C-26), 24.79(C-27), 182.8(C-28), 27.3(C-29), 16.6(C-30).

**2α-hydroxyl ursolic acid (4)**, white powder. ^1^H NMR(600 MHz, CD_3_OD) *δ*: 2.91(1H, d, *J*=9.6 Hz, H-3), 5.24(1H, t, *J*=3.7 Hz, H-12), 2.20(1H, dd, *J*=11.6, 1.8 Hz, H-18), 1.13(3H, s, H-23), 0.85(3H, s, H-24), 0.81(3H, s, H-25), 1.02(6H, brs, H-26, 30), 0.97(3H, s, H-27), 0.89(3H, d, *J*=6.5 Hz, H-29), 1.02(3H, s, H-30); ^13^C NMR(150 MHz, CD_3_OD) *δ*: 48.3(C-1), 69.5(C-2), 84.5(C-3), 40.8(C-4), 56.7(C-5), 19.5(C-6), 34.2(C-7), 40.5(C-8), 49.6(C-9), 39.2(C-10), 24.5(C-11), 126.7(C-12), 139.7(C-13), 43.3(C-14), 29.2(C-15), 25.3(C-16), 48.1(C-17), 54.4(C-18), 40.4(C-19), 40.4(C-20), 31.8(C-21), 38.1(C-22), 29.2(C-23), 17.8(C-24), 17.2(C-25), 17.6(C-26), 24.1(C-27), 181.6(C-28), 17.5(C-29), 21.6(C-30).

**Apigenin-7-O-rutinoside (5)**, yellow powder. ^1^H NMR(600 MHz, DMSO-*d_6_*) *δ*: 6.86(1H, s, H-3), 12.95(1H, s, 5-OH), 6.45(1H, d, *J*=2.1 Hz, H-6), 6.77(1H, d, *J*=2.2 Hz, H-8), 7.95(2H, d, *J*=8.7 Hz, H-2′, 6′), 6.95(2H, d, *J*=8.8 Hz, H-3′, 5′), 10.38(1H, s, 4′-OH), 5.06(1H, d, *J*=7.5 Hz, glc-1), 4.55(1H, d, *J*=1.6 Hz, rha-1), 1.07(3H, d, *J*=6.2 Hz, rha-6); ^13^C NMR(150 MHz, DMSO-*d_6_*) *δ*: 164.4(C-2), 103.1(C-3), 182.1(C-4), 161.2(C-5), 99.9(C-6), 162.9(C-7), 94.8(C-8), 156.9(C-9), 105.4(C-10), 121.1(C-1′), 128.7(C-2′, 6′), 116.1(C-3′, 5′), 161.3(C-4′), 99.5(glc-1), 73.1(glc-2), 76.3(glc-3), 69.6(glc-4), 75.6(glc-5), 66.1(glc-6), 105.5(rha-1), 70.4(rha-2), 70.8(rha-3), 72.1(rha-4), 68.4(rha-5), 17.8(rha-6).

**Rosmarinic acid (6)**, yellow and oily liquid. ^1^H NMR(600 MHz, DMSO-*d_6_*) *δ*: 7.06(1H, d, *J*=2.1 Hz, H-2), 6.77(1H, d, *J*=8.1 Hz, H-5), 7.01(1H, dd, *J*=8.2, 2.0 Hz, H-6), 7.46(1H, d, *J*=15.9 Hz, H-7), 6.25(1H, d, *J*=15.9 Hz, H-8), 6.69(1H, d, *J*=2.1 Hz, H-2′), 6.53(1H, dd, *J*=8.1, 2.0 Hz, H-5′), 6.64(1H, d, *J*=8.0 Hz, H-6′), 3.0(1H, m, H-7′a), 2.9(1H, m, H-7′b), 5.03(1H, dd, *J*=8.6, 4.1 Hz, H-8′); ^13^C NMR(150 MHz, DMSO-*d_6_*) *δ*: 125.4(C-1), 113.5(C-2), 145.7(C-3), 148.7(C-4), 115.9(C-5), 121.7(C-6), 145.9(C-7), 115.0(C-8), 166.0(C-9), 127.6(C-1′), 116.8(C-2′), 145.0(C-3′), 144.1(C-4′), 115.5(C-5′), 120.1(C-6′), 36.3(C-7′), 73.2(C-8′), 171.1(C-9′).

**Methyl rosmarinate (7)**, yellow and oily liquid. ^1^H NMR(600 MHz, DMSO-d6) *δ*: 7.09(1H, s, H-2), 6.78(1H, s, H-5), 7.01(1H, d, *J*=7.6 Hz, H-6), 7.48(1H, d, *J*=15.9 Hz, H-7), 6.26(1H, d, *J*=15.9 Hz, H-8), 6.66(1H, s, H-2′), 6.49(1H, d, *J*=7.3 Hz, H-5′), 6.64(1H, s, H-6′), 2.96(2H, m, H-7′), 5.11(1H, dd, *J*=7.3, 5.3 Hz, H-8′), 3.63(3H, s, -OCH_3_); ^13^C NMR(150 MHz, DMSO-*d_6_*) *δ*: 125.2(C-1), 112.8(C-2), 145.8(C-3), 149.1(C-4), 115.5(C-5), 121.7(C-6), 146.5(C-7), 115.1(C-8), 166.0(C-9), 126.6(C-1′), 116.8(C-2′), 145.1(C-3′), 144.3(C-4′), 115.9(C-5′), 120.1(C-6′), 36.3(C-7′), 72.8(C-8′), 170.0(C-9′), 52.3(-OCH_3_).

**Luteolin (8)**, orange powder. ^1^H NMR(600 MHz, DMSO-*d_6_*) *δ*: 6.65(1H, s, H-3), 12.97(1H, s, 5-OH), 6.18(1H, s, H-6), 6.43(1H, s, H-8), 7.39(1H, s, H-2′), 6.88(1H, d, *J*=8.1 Hz, H-5′), 7.41(1H, m, H-6′); ^13^C NMR(150 MHz, DMSO-*d_6_*) *δ*: 163.9(C-2), 102.8(C-3), 181.6(C-4), 161.5(C-5), 98.9(C-6), 164.8(C-7), 93.9(C-8), 157.3(C-9), 103.6(C-10), 121.4(C-1′), 113.3(C-2′), 145.8(C-3′), 149.9(C-4′), 116.0(C-5′), 119.0(C-6′).

**Diosmetin (9)**, orange powder. ^1^H NMR(600 MHz, DMSO-*d_6_*) *δ*: 6.72(1H, s, H-3), 12.92(1H, s, 5-OH), 6.18(1H, s, H-6), 6.44(1H, s, H-8), 7.42(1H, s, H-2′), 7.07(1H, d, *J*=8.5 Hz, H-5′), 7.52(1H, m, H-6′), 3.86(3H, s, 4′-OCH_3_); ^13^C NMR(150 MHz, DMSO-*d_6_*) *δ*: 163.4(C-2), 103.6 (C-3), 181.6(C-4), 157.4(C-5), 99.1(C-6), 164.9(C-7), 94.0(C-8), 161.5(C-9), 103.5(C-10), 118.7(C-1′), 112.9(C-2′), 146.8(C-3′), 151.1(C-4′), 112.1(C-5′), 123.1(C-6′), 55.8(4′-OCH_3_).
